# Persistent foramen ovale closure in divers with a history of decompression sickness

**DOI:** 10.1007/s12471-018-1153-x

**Published:** 2018-09-03

**Authors:** R. Koopsen, P. R. Stella, K. M. Thijs, R. Rienks

**Affiliations:** 1 Utrecht University, Utrecht, The Netherlands; 2 Department of Cardiology, University Medical Center, Utrecht, The Netherlands; 3 Department of Sports Medicine, University Medical Center, Utrecht, The Netherlands; 4 Central Military Hospital, Utrecht, The Netherlands

**Keywords:** Patent foramen ovale, Decompression sickness, Diving

## Abstract

**Objective:**

To study the effect of percutaneous patent foramen ovale (PFO) closure in divers with a history of decompression sickness (DCS).

**Study design:**

(1) Retrospective study of patient records and (2) telephonic follow-up. Patients with unexplained decompression sickness, who were referred to a cardiologist with a focus on diving medicine between 2000 and 2017, were included in the study

**Results:**

A total of 62 divers with DCS were included. In all cases transoesophageal echocardiography (TEE) was performed, showing 29 PFOs and 6 atrial septum defects (ASDs) in total *n* = 35 (56%). The highest prevalence was found in divers with cutaneous and vestibular DCS. At follow-up (mean follow-up duration 6.8 years), 21 PFOs/ASDs were closed using a percutaneous procedure. One diver was lost to follow-up. One diver quit diving. The remaining divers were able to resume unrestricted diving; there was no recurrence of major DCS. Of the divers with an open PFO or ASD, 14 were included of whom 7 are currently diving. All (except one diver with a small PFO) divers are using a conservative diving profile to reduce nitrogen load and the appearance of venous nitrogen bubbles. There was no recurrence of major DCS in this group.

**Conclusion:**

Percutaneous PFO closure may be an effective and safe treatment for divers who have suffered a major DCS to return to unrestricted diving. Alternatively, conservative treatment seems safe when divers refrain from unrestricted diving and use a conservative technique in order to reduce nitrogen load.

## What’s new?

- Vestibular DCS and cutis marmorata are frequently associated with PFO.
- PFO closure in divers with a history of DCS is a safe and effective treatment for divers to return to unrestricted diving.
- If the diver refrains from PFO closure, a restrictive diving profile seems safe and could be recommended.

## Introduction

Decompression illness (DCI) is a major medical problem among scuba divers. As a diver descends and breathes air under increased pressure, the tissues become loaded with increased quantities of nitrogen. During ascent, but especially after the dive, this nitrogen is released into the blood which can lead to bubble formation. The volume and location of these bubbles determine whether symptoms occur [1].

Decompression illness (DCI) comprises both arterial gas embolism (AGE) and decompression sickness (DCS). AGE usually occurs directly after surfacing and is caused by lung problems (bullae or blebs) or a provocative diving profile (rapid ascent with breath holding), causing overstretching and tearing of the alveoli leading to air bubbles in the pulmonary veins and subsequently into the arterial system (pulmonary barotrauma).

DCS is caused by nitrogen bubbles. The nitrogen that has been stored in the tissues during the dive disappears by diffusion into the venous system. It is subsequently transported to the lungs and exhaled. When the pressure decreases during ascent, and especially after the dive, there may be bubble formation in the tissue and in the veins. This occurs when the nitrogen concentration exceeds the capacity of the tissue and the venous system to remove the nitrogen. Local bubble formation usually results in ‘type 1’ DCS whereas venous bubble formation may result in ‘type 2’ DCS in the presence of a right-to-left shunt. DCS usually develops from about 20 min after surfacing.

‘Minor decompression illness’, or *type 1* manifests as musculoskeletal and cutaneous symptoms, such as pain around the joint, skin rash and pruritus. ‘Major decompression illness’, or *type 2* causes neurological, vestibular or pulmonary symptoms [2].

Recently it was hypothesised that cutis marmorata, which had previously been classified under type 1 DCS, may be better classified under type 2 DCS, because of its postulated neurogenic origin [3, 4].

When DCI occurs when diving safety limits have been violated it is considered to be ‘deserved’ DCI. However, in half of the cases, DCI is ‘undeserved’ i. e. it occurs despite compliance with the standardised decompression procedures. In cases of undeserved DCI, it is warranted to look for a right-to-left shunt facilitating the transition of nitrogen bubbles from the venous to the arterial circulation. The most frequent cause of a right-to-left shunt is patent foramen ovale (PFO). Although PFO is present in a quarter to one-third of the general population, the risk of having a DCI event is low and has been reported to be 2.5 in 10,000 divers. However it is five times higher in divers with a PFO than in those without a PFO [5]. It is suggested that a small PFO may not contribute to DCI, and a large PFO (diameter >10 mm) may [6].

There are few data on the recurrence of decompression sickness after the first event. In a group of 50 recreational divers with neurological DCS, neurological DCS reoccurred in 48% of the divers [7]. A prospective study in 104 scuba divers (divided into 3 groups: no PFO, closed PFO and open PFO) performing 18,394 dives over a period of 5 years showed major DCI events mainly in the open PFO group, suggesting a protective effect of PFO closure [8]. Another study comparing divers with a PFO to divers treated with catheter-based PFO closure showed complete elimination of arterial bubbles after simulated dives [9].

Percutaneous closure of PFO has been used to reduce the risk of DCI and has been shown to be safe and allow most patients to return to unrestricted diving [10]. However, the effect of closure on the rate of recurrence of DCI has not been properly established. Our study observed the clinical course of patients who had suffered a DCS, whether or not after PFO closure.

## Methods

This retrospective study was conducted at the University Medical Center Utrecht. Patients with unexplained decompression sickness, who were referred to a cardiologist with a focus on diving medicine between 2000 and 2017, were included in the study.

Data were retrieved from our electronic patient file. At time of presentation, a detailed description of the diving accident, timetable of occurrence of symptoms, treatment, medical history and diving experience were obtained. A DCS event was classified into different types of DCS: musculoskeletal, skin bends, cutis marmorata, neurological or vestibular.

Analysis at presentation included transoesophageal echocardiography (TEE) with contrast and using the Valsalva manoeuver for detecting right-to-left shunts, computed tomography (CT) scan of the thorax and magnetic resonance imaging (MRI) of the cerebrum.

A telephone questionnaire was used to gather information about current diving activities. The divers were asked if they were currently diving, if they had changed their diving profile focusing on reduction of venous bubble load (use of nitrox, restrictions on the depth of the dive, strict adherence to decompression tables, no repetitive dives during a single day and reduced rate of ascent) and if they had experienced any diving-related problems such as DCS.

All PFO and atrial septal defects (ASD) closures were performed using a standardised procedure as has been described previously [11]. The PFO/ASD was closed under local anaesthesia with intracardiac echocardiography (ICE) guidance. In almost 90% of the cases a Figulla Flex PFO occluder was used (Occlutech) while the remaining 10% was closed with an Amplatzer PFO occluder (ABBOTT). Post-procedural thrombosis prophylaxis consisted of 6 months of treatment with carbasalate calcium and 3 months of clopidogrel. The advice was given to resume diving six months after the procedure. At that time, the antiplatelet therapy was discontinued because it is presumed that full endothelialisation of the atrial wall has taken place [12].

## Results

A total of 77 divers were referred to our hospital between 2000 and 2017 for cardiac analyses after a decompression illness. Ten divers were excluded because decompression illness was unlikely, one diver did not attend the examination. Another four divers were excluded because they suffered from arterial gas embolism (AGE); 62 divers were analysed with a history of decompression sickness.

In all cases, a transoesophageal echocardiography (TEE) was performed, showing 29 PFOs and 6 haemodynamically not significant ASDs (*n* = 35 in total: 56%). No other right-to-left shunts were found. The echocardiographic data are presented in Tab. 1. We found a high prevalence of PFO in the subgroup of divers with cutis marmorata (80%) and vestibular DCS (82%). A PFO or ASD was only found in 35% of divers with neurological DCS.

**Table 1:** Patient characteristics and analysis of type of DCS and prevalence of PFO

DCS	Total (*n* = 62)	PFO/ASD absent (*n* = 27)	PFO/ASD present (*n* = 35)	PFO/ASD closed (*n* = 21)	PFO/ASD not closed (*n* = 14)
Male (%)	58.1	66.7	51.4	57.1	42.9
Mean age (years, range)	38.3 (20–61)	33.0 (20–55)	42.3 (25–61)	44.7 (25–61)	38.8 (30–61)
*Type 1*					
Musculoskeletal	2	2	0	0	0
Skin bends	8	3	5	2	3
*Type 2*					
Cutis marmorata	15	3	12	10	2
Neurological	26	17	9	3	6
Vestibular	11	2	9	6	3

*ASD* atrial septum defect, *DCS* decompression sickness, *PFO* patent foramen ovale

**Fig. 1:**
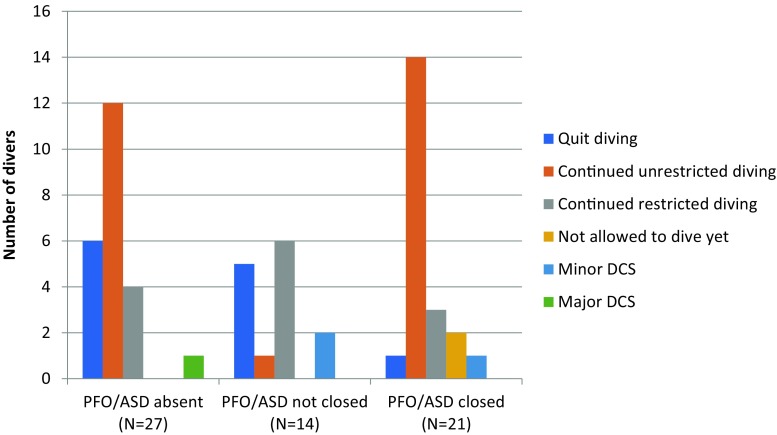
Clinical follow-up

The mean follow-up duration was 6.8 years (range 0.5–11.6 years). At follow-up, there were 14 divers with an open PFO. Two of them were lost to follow-up. Four divers chose to change their diving profile instead of PFO closure in order to reduce venous bubble load, and continued diving. In two divers, closure of the PFO was not offered because they had a history of skin bends, which was later not considered to be an indication for percutaneous closure of an PFO. They continued restricted diving as well. Three divers had other strict contraindications for diving such as pulmonary bullae or pulmonary emphysema, which made closing of the PFO irrelevant. One diver decided to quit diving. One diver, with a minor PFO, continued unrestricted diving. No recurrence of major DCS occurred. Two divers experienced minor DCS during follow-up, one with an unrestricted diving profile and one with restrictions (Fig. 1).

In 21 divers the PFO or ASD was closed, one of whom was lost to follow-up. Out of 20 divers, 17 continued diving. Three had changed their diving profile additionally. In two divers the PFO had been closed recently and they were not yet allowed to dive. None of the divers experienced recurrence of major DCS after the PFO was closed. However, one diver experienced minor DCS/skin bends after PFO closure. Four divers spontaneously reported that, when looking retrospectively, they might have experienced multiple minor DCS in the past as well, before the PFO was closed, which they had not recognised as such at the time. One diver was no longer diving despite successful closure of a PFO.

In one diver, paroxysmal atrial fibrillation occurred directly after closure of the PFO. A TTE with contrast, at 4‑month follow-up, did not show any residual shunts among the treated divers.

Most divers with no cardiac pathology continued diving without adjusting their diving profile (55%). In one subject, recurrence of DCS occurred.

## Discussion

This study attempted to establish the efficacy of PFO closure in patients with a history of DCS. In 20 divers with a closed PFO, major DCS did not reoccur. All divers were able to go back to unrestricted diving, although three divers preferred to continue their diving activities more conservatively. As the present study is descriptive and there is no comparison group, the efficacy of PFO closure could not be statistically quantified.

Conservative or restricted diving is generally based on common-sense advice with regard to decompression safety. Clear guidelines on restricted diving are lacking. Klingmann et al. observed a significant reduction of DCS after providing the following recommendations: use of nitrox with decompression times calculated on air tables, no deep dives (>25 meters), no repetitive dives, minimisation of Valsalva manoeuvres, no decompression dives and a 5-minute safety stop at 3 msw [13].

Currently, the most frequent indication for PFO closure is the increased risk of cryptogenic stroke, migraine and vascular headache. The safety and efficacy of percutaneous closure of PFO has been the subject of several studies. Three multicentre studies comparing PFO closure to antiplatelet therapy have shown the safety of PFO closure. These trials found a significantly higher rate of (transient) atrial fibrillation in the PFO group than in the comparison group but no differences in serious adverse events [14–16]. In our population, paroxysmal atrial fibrillation occurred in one patient.

Since the majority of the group with an open PFO either quit diving or changed to a more conservative diving profile, it is not possible to compare the group with an open PFO to the group with a closed PFO with respect to unrestricted diving. However, in this group no recurrence of major DCS was seen either.

In the group without PFO or ASD, major DCS reoccurred in one subject, which was later attributed to the complications of sinusitis.

Billinger et al. found no recurrences of major DCI during long-term follow-up in 26 divers (1,208 dives) who underwent percutaneous closure of their PFO, which is consistent with our findings. Yet, a case report has been published which describes a diver who redeveloped cutaneous DCI after treatment with a PFO occluder four years earlier [17].

The overall prevalence of right-to-left shunts in this population of divers with undeserved DCS is 56%. This is higher than expected in the general population (approximately 27%) [5]. We found a high prevalence of right-to-left shunts in both divers with cutis marmorata (80%) and in divers with vestibular DCS (82%).

In the present patient group, initially skin bends and cutis marmorata were considered equally as ‘cutaneous DCS’, and thus ‘minor DCS’. There is increasing interest in the aetiology of cutaneous DCS. Kemper et al. proposed that cutis marmorata may be cerebrally mediated, as they found an association between cutis marmorata and cardiac right-to-left shunts [4]. This is consistent with our high prevalence of shunts in this population. Also, this new insight can explain why originally no clear policy was followed regarding PFO closure in patients with a history of skin bends. At first, the PFOs of two divers were closed, later two divers were advised to use a more conservative diving profile.

In comparison to cutis marmorata and vestibular DCS, we found a relatively low prevalence of right-to-left shunts in patients with a neurological DCS (38%). A likely explanation may be that patients with unspecific symptoms such as fatigue, weakness, nausea, headache and disorientation were all classified as neurological DCS. However, we did not find a high prevalence of PFO/ASD in the patients with more specific neurological symptoms. In our population, no cases of paralysis or paraplegia occurred.

## Conclusion

**Fig. 2:**
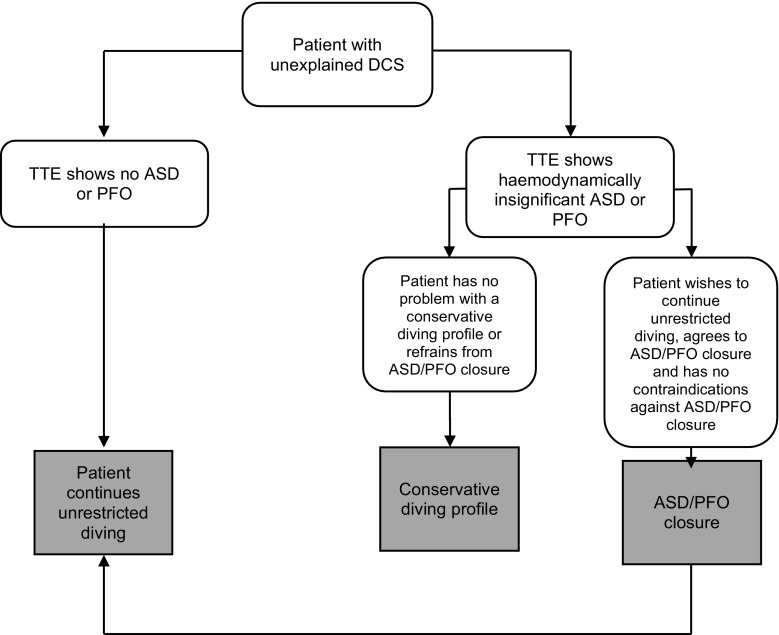
Clinical decision making

For divers who had suffered a major DCS, percutaneous PFO closure may be an effective and safe treatment to return to unrestricted diving. Alternatively, a restrictive diving profile seems safe and could be recommended for divers who refrain from PFO closure (Fig. 2).

## References

[1] Baratt DM, Harch PG, Van Meter K (2002). Decompression illness in divers: a review of the literature. Neurologist.

[2] Lynch JH, Bove AA (2009). Diving medicine: a review of current evidence. J Spec Oper Med.

[3] Wilmshurst PT, Pearson MJ, Walsh KP, Morrison WL, Bryson P (2001). Relationship between right-to-left shunts and cutaneous decompression illness. Clin Sci.

[4] Kemper TC, Rienks R, van Ooij PJ, van Hulst RA (2015). Cutis marmorata in decompression illness may be cerebrally mediated: a novel hypothesis on the aetiology of cutis marmorata. Diving Hyperb Med.

[5] Torti SR, Billinger M, Schwerzmann M (2004). Risk of decompression illness among 230 divers in relation to the presence and size of patent foramen ovale. Eur Heart J.

[6] Wilmshurst PT, Morrison WL, Walsh KP (2015). Comparison of the size of persistent foramen ovale and atrial septal defects in divers with shunt-related decompression illness and in the general population. Diving Hyperb Med.

[7] Gempp E, Louge P, Blatteau JE, Hugon M (2012). Risks factors for recurrent neurological decompression sickness in recreational divers: a case-control study. J Sports Med Phys Fitness.

[8] Billinger M, Zbinden R, Mordasini R (2011). Patent foramen ovale closure in recreational divers: effect on decompression illness and ischaemic brain lesions during long-term follow-up. Heart.

[9] Honěk J, Srámek M, Sefc L (2014). Effect of catheter-based patent foramen ovale closure on the occurrence of arterial bubbles in scuba divers. JACC Cardiovasc. Interv..

[10] Pearman A, Bugeja L, Nelson M, Szantho GV, Turner M (2015). An audit of persistent foramen ovale closure in 105 divers. Diving Hyperb Med.

[11] Wilmshurst P, Walsh K, Morrison L (1999). Transcatheter closure of patent foramen ovale using the Amplatzer septal occluder to prevent recurrence of neurological decompression illness in divers. Heart.

[12] Sigler M, Jux C (2007). Biocompatibility of septal defect closure devices. Heart.

[13] Klingmann C, Rathmann N (2012). Hausmann et al. Lower risk of decompression sickness after recommendation of conservative decompression practices in divers with and without vascular right-to-left shunt. Diving Hyperb Med.

[14] Mas J, Derumeaux G, Guillon B (2017). Patent foramen ovale closure or anticoagulation vs. antiplatelets after stroke. N Engl J Med.

[15] Sondergaard L, Kasner S, Rhodes J (2017). Patent foramen ovale closure or antiplatelet therapy for cryptogenic stroke. N Engl J Med.

[16] Saver J, Carroll J, Thaler D (2017). Long-term outcomes of patent foramen ovale closure or medical therapy after stroke. N Engl J Med.

[17] Vande Eede M (2016). Recurrent cutaneous decompression illness after PFO device implantation: a case report. Undersea Hyperb Med.

